# Authors’ Reply: A comparison of different methods to handle missing data in the context of propensity score analysis

**DOI:** 10.1007/s10654-019-00553-y

**Published:** 2019-08-27

**Authors:** Jungyeon Choi, Olaf M. Dekkers, Saskia le Cessie

**Affiliations:** 1 Department of Clinical Epidemiology, Leiden University Medical Center, Albinusdreef 2, C7-P, 2333 ZA Leiden, The Netherlands; 2 Department of Clinical Epidemiology and Department of Endocrinology and Metabolism, Leiden University Medical Center, Albinusdreef 2, C7-P, 2333 ZA Leiden, The Netherlands; 3 Department of Clinical Epidemiology and Department of Biomedical Data Sciences, Leiden University Medical Center, Albinusdreef 2, C7-P, 2333 ZA Leiden, The Netherlands

We would like to thank Choi and Gelfond [1] for a critical review regarding our study which compares different methods to handle missing data in the context of propensity score analysis. The authors pointed out that the simulation scenarios in our study are limited for two reasons:

1. A scenario where missingness of covariates is dependent of the outcome was not considered.
2. A non-null treatment effect under homogenous treatment effect was not considered.

In this Reply we will discuss the reason why we left out a scenario where missingness is dependent of the outcome and show that our simulation results are consistent when there is a non-null treatment effect.

**Fig. 1:**
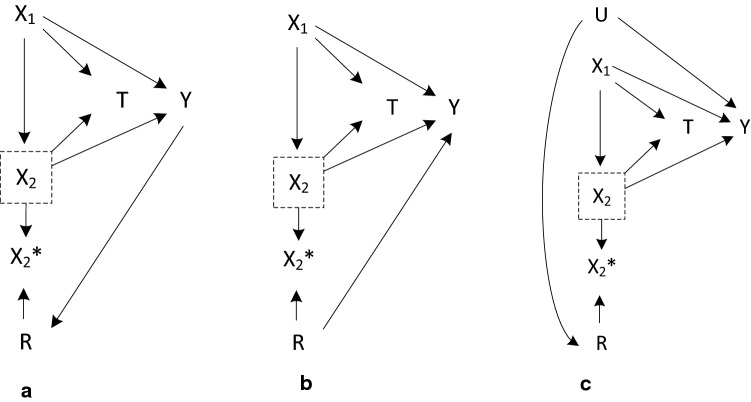
M-graphs for situations where missingness in a covariate (R) cannot be d-separated with the outcome (Y)

Regarding the first point, we agree with the authors that a complete case analysis will results in bias when data are missing at random and the missingness in covariates is dependent on the outcome. This can also be shown in a missingness graph (Fig. 1a). In this scenario, a complete case analysis will result in bias since the missing indicator R and the outcome Y cannot be d-separated. In fact, complete case analysis will result in bias in any situation when missingness in covariates (R) and the outcome (Y) cannot be conditionally d-separated even when the treatment effect is homogeneous. This can happen in three different scenarios:

(i) Y has a direct effect on R (Fig. 1a)
(ii) R has a direct effect on Y (Fig. 1b)
(iii) R and Y is indirectly associated by an unobserved common cause (Fig. 1c)

Scenario (i), an issue raised by Choi et al., was intentionally left out since we were doubtful how often such scenario would occur in real clinical settings. The idea of propensity score methods is that a treatment assignment is made based on a patient’s baseline characteristics. Therefore, balancing baseline covariates of two treatment groups would lead to exchangeability between the groups conditional on the propensity scores; which is why the method is often described as “mimicking a randomized control trial”. Keeping this in mind, it is difficult to come up with a real clinical situations where missingness in baseline variables (which are observed before the treatment assignment) are dependent on the outcome (which is observed only after a treatment is given). Situations like scenario (i) may occur in cross-sectional studies where all variables are collected at the same moment. Although the technique of propensity score methods can be used in analyzing cross-sectional data, it is uncommon to use a cross-sectional design for estimating a causal treatment effect.

Scenario (ii) implies that whether a certain covariate is measured or not has an effect on patients prognosis which we also found unlikely to happen in clinical settings and therefore has not been included in our simulation scenarios. Scenario (iii), which may more commonly occur in clinical settings, has been addressed in Simulation Setting 3 (with an additional arrow from U to X_2_).

In simulation studies, limited amount of simulation scenarios may substantially affect the validity of a study. At the same time, it is important to keep in mind how real life clinical settings are constructed. Countless hypothetical scenarios can be generated, but few may have clinical relevance.

Regarding the second point raised by Choi et al., it is indeed true that conditional exchangeability in the R = 0 group, which can be written as:

1 $$ (Y1,Y0)\, \bot \,Z\,|\,X,R = \, 0, $$

should hold for a propensity score analysis with complete cases to yield a valid estimate of the ATE for the R = 0 group. For the treatment effect estimated from condition (1) to be an unbiased estimates of the average marginal treatment effect in the population, the treatment effect in the partially observed individuals (R = 1) should be equal to that of the fully observed individuals (R = 0). This implies that there should be no heterogenous treatment effect between the two groups, which can be written as the condition stated in our paper:

2 $$ E[Y1 - Y0\,|\,R = 0] = E[Y1 - Y0\,|\,R = 1] = E[Y1 - Y0] $$

That complete case analysis no longer yields valid estimates of the marginal population effects when the treatment effect is heterogeneous has been shown in the results of our paper in Simulation setting 2. We agree with the comment made by Choi et al. that one should be aware that the homogeneity of treatment effect assumption may depend on the type of estimand (e.g. using relative risks versus risk differences).

**Fig. 2:**
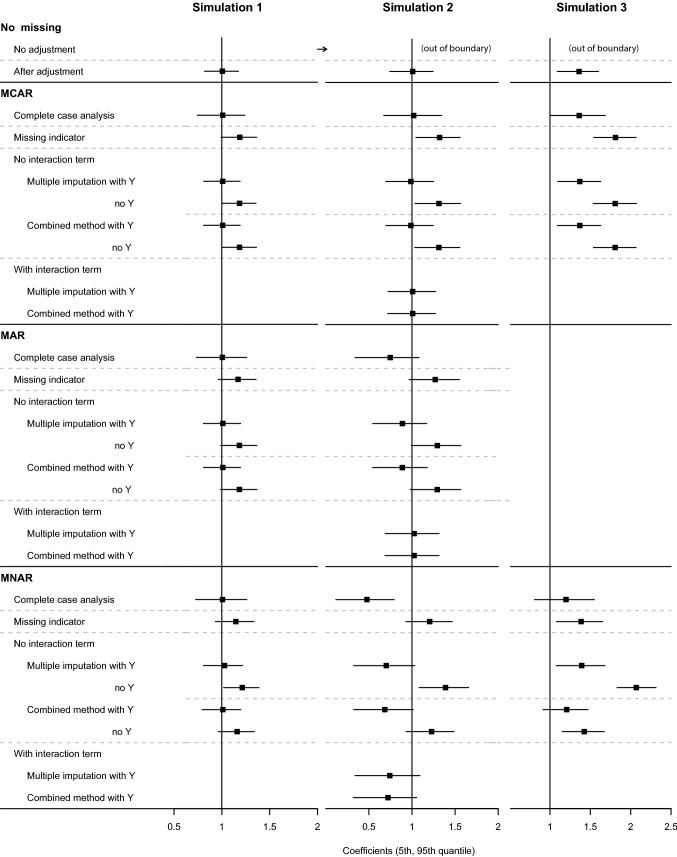
Mean treatment effects and their 5th and 95th percentile ranges estimated by propensity weighting in Simulation setting 1 (left), 2 (middle) and 3 (right) with a non-null treatment effect

The conditions (1) and (2) do not require a null treatment effect. We ran our simulation setting 1, 2 and 3 again with a non-null treatment effect. The average treatment effect was set to be 1 while all other parameters for data generation were kept equal to the previous simulations. The treatment effect is homogenous in Simulation setting 1 and 3. Figure 2 shows the results of 1000 simulation runs. The results are consistent to the results of our main paper.
